# Antitumour, Antimicrobial, Antioxidant and Antiacetylcholinesterase Effect of *Ganoderma Lucidum* Terpenoids and Polysaccharides: A Review

**DOI:** 10.3390/molecules23030649

**Published:** 2018-03-13

**Authors:** Darija Cör, Željko Knez, Maša Knez Hrnčič

**Affiliations:** 1 Faculty of Chemistry and Chemical Engineering, University of Maribor, SI-2000 Maribor, Slovenia; darija.cor@um.si (D.C.); zeljko.knez@um.si (Ž.K.); 2 Faculty of Medicine, University of Maribor, SI-2000 Maribor, Slovenia

**Keywords:** biological activity, triterpenoids, polyscaccharides, *G. lucidum*

## Abstract

*Ganoderma lucidum* (Reishi) is a popular medicinal mushroom and has been used in oriental medicine because of its promoting effects on health and life expectancy. *G. lucidum* contains various compounds with a high grade of biological activty, which increase the immunity and show antitumour, antimicrobial, anti-inflammatory, antioxidant and acetylcholinesterase inhibitory activity. Several of these substances belong to the triterpenoids and polysaccharides classes. Proteins, lipids, phenols, sterols, etc. are also present. In the present review, an extensive overview of the presence of antitumour, antimicrobial, antioxidant and antiacetylcholinesterase compounds in *G. lucidum* extracts will be given, along with an evaluation of their therapeutic effects.

## 1. Introduction

Reishi or Lingzhi is the Japanese or Chinese name for *Ganoderma lucidum* (*G. lucidum*) (Curtis: Fr.) P. Karst, a woody Basidiomycetes mushroom belonging to the family of Ganodermaceae of the Aphyllophorals. In Nature, it can be mostly found growing on living and dead wood of deciduous species under high humidity and indistinct lighting conditions [1]. It is a saprophyte or facultative parasite. In Nature, it grows in the subtropical and temperate climate zones, in the forests of Asia, Europe and North and South America [2].

Reishi has been widely used in traditional Chinese medicine for promoting health, longevity and spiritual growth [3]. The name Reishi was first recognized more than 2400 years ago by the herbalist Shen Nong from the Shu Dynasty and it was then classified as a “superior herb”, which means that it can be taken constantly without any side effects [4]. It has been used for the prevention as well as the treatment of various diseases, such as chronic hepatitis, nephritis, high blood pressure, bronchitis and tumorigenic afflictions [5] since ancient time and is a super immune stimulant, which strongly protects the entire body.

Amongst the more than 2000 classes of Reishi, known to date, only six them—red, black, blue, white, yellow and purple Reishi—have been investigated to discover potential health-beneficial properties. Among them, black Reishi (*G. sinensis*) and red Reishi (*G. lucidum*) have shown the most significant health-enhancing effects [1].

It has been reported that *G. lucidum* “the mushroom of immortality” yields miraculous health benefits and contains over 400 bioactive compounds, including triterpenoids, polysaccharides, nucleotides, sterols, steroids, fatty acids and proteins/peptides, which have a number of medicinal effects [6,7] like anti-tumour [8,9], anti-microbial [10], anti-atherosclerotic [11], anti-inflammatory, hypolipidemic [12], anti-diabetic, anti-oxidative and radical-scavenging, anti-aging [13], anti-fungal, and anti-viral (specifically against herpes and HIV) effects, as well as boosting the immune system. The most important pharmacologically active compounds are triterpenoids and polysaccharides [14]. 

**Figure 1:**
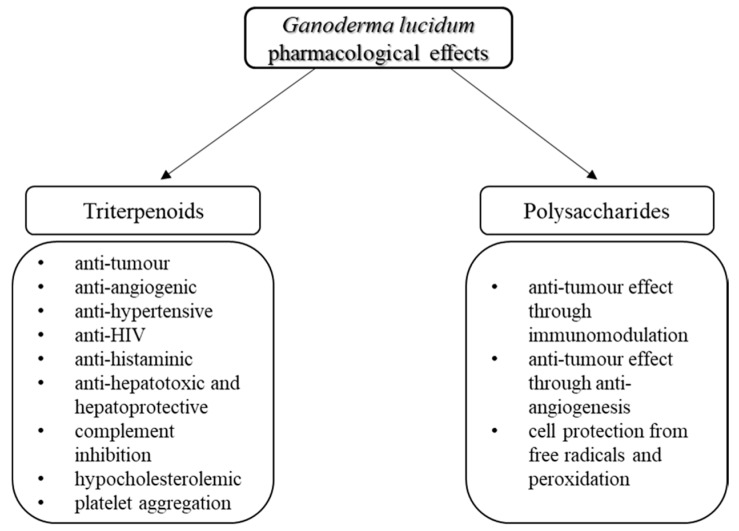
*Ganoderma lucidum* pharmacological effects related to the specific group of biological compounds [14].

**Figure 2:**
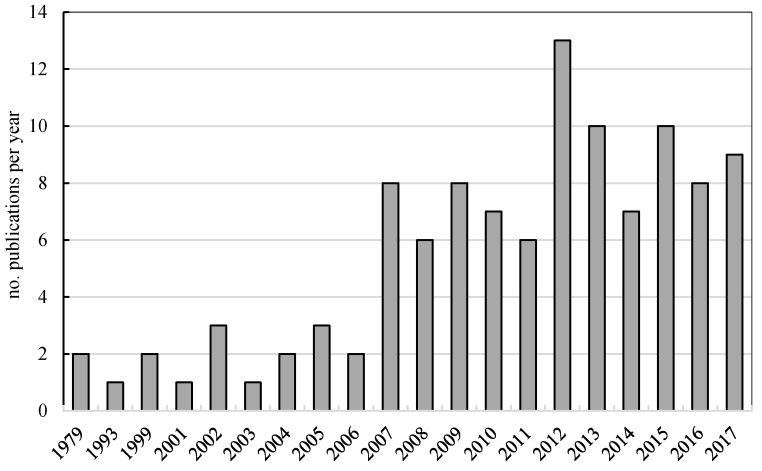
Distribution of publications per year from Scopus Database according to the keyword search: “*Ganoderma lucidum* pharmaceutical” [15].

Research has now confirmed that *G. lucidum* induces a self-triggered immune response and is a very powerful antioxidant. Nowadays it is also being used in modern medicine as a supplement to cancer treatment and to fight the side-effects of chemotherapy in China and also in Western countries [4]. Figure 1 shows the most common pharmacological effects of the triterpenoids and polysaccharides isolated from *G. lucidum*. Figure 2 gives a distribution of publications on this topic by year. Including 1979, overall about 179 scientific papers were found with the keywords “*Ganoderma lucidum* pharmaceutical” in the Scopus database [15].

The present review presents this miraculous medicinal mushroom *G. lucidum* along with the antitumour, antimicrobial, antioxidant and antiacetylcholinesterase effects of its various components.

## 2. Composition of *G. lucidum*

The majority of the mushroom’s weight is derived from its high water content, ranging up to 90%, which makes a basic mushroom ‘extract’ dehydrated mushroom powder (and thus 1 g of extract, if unspecified, may be about as strong as 10 g of the mushroom). Elsewhere, the residual 10% of its mass consists of protein (10–40%), fat (28%), carbohydrate (3–28%), fibre (3–32%), ash (8–10%,). Other complex compounds like pro-vitamin D2 [16], C19 fatty acids [17] and essential nutrient metals such as copper [18], zinc [18] and selenium [19] were also detected. Table 1 proves that potassium, calcium, phosphorus, magnesium, selenium, iron, zinc, and copper represent most of the mineral content [20,21]. Furthermore, *G. lucidum* possesses a wide variety of bioactive molecules like terpenoids, steroids, phenolic compounds, nucleotides and their derivatives, different carbohydrates including glycoproteins and polysaccharides. The mushroom proteins contain different essential amino acids. Leucine and lysine are present in notably high percentages. *G. lucidum* also has a high proportion of polyunsaturated fatty acids compared to the total fatty acids in mushrooms, which are substantial contributors to the high health importance [6,20].

### Bioactive Molecules Found in G. lucidum

*G. lucidum* contains a large quantity of unique bioactive molecules such as polysaccharides and triterpenoids. Triterpenoids have been reported to have anti-hypertensive, hypocholesterolemic, hepatoprotective, and anti-histaminic effects, as well as antitumour and anti-angiogenic activity. Polysaccharides, particularly β-d-glucans, have been known to possess antitumour effects. Furthermore, polysaccharides have a shielding effect against free radicals and decrease cell harm triggered by mutagens [13].

**Figure 3:**
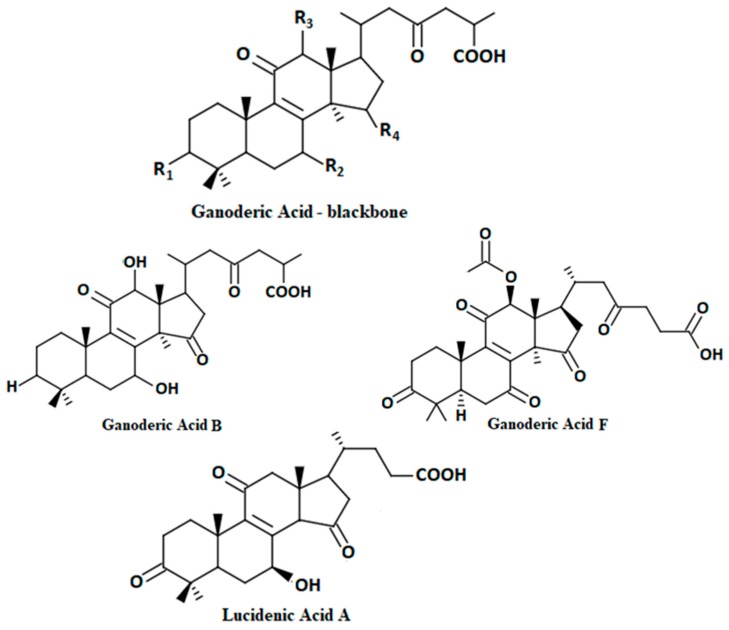
Structural formulas of ganoderic acid and lucidenic acid [23].

A variety of polysaccharides and triterpenoids results in several biological activities of *G. lucidum* [23]. The cell walls of *G. lucidum* spores contain a high number of polysaccharides. A variety of bioactive polysaccharides isolated from *G. lucidum* have been found to be complex β-1,3-glucans polysaccharide peptides like peptidoglycan, which interact with the immune system. Several water-soluble polysaccharides have been fractionated and purified from the aqueous extract of *G. lucidum* [24,25,26,27,28,29]. Over 140 triterpenoid compounds were found in *G. lucidum* extracts, which can be separated into ganoderma acids or ganoderma alcohols. Some of them are presented in Figure 3 [23,30]. Some triterpene-rich extracts of *G. lucidum* contain high amounts of lucidenic acids which can purified from the extract, and exert an immunostimulatory function [31,32]. Several nucleotides and nucleobases were qualitatively identified in the mushroom samples [33]. 

## 3. Bioactivity of *G. lucidum* Triterpens

Triterpenes are a subtype of terpenes and are widely distributed throughout the plant world. As a species of pharmaceutical substances, they contribute to the biological ability of *G. lucidum*. It has been proven that many of these triterpenoids, especially the ganoderic acids (GAs), are bioactive components [3,34,35,36] responsible for several biological effects, including anti-inflammatory [35], anti-tumourigenic [36], anti-HIV [37] and hypolipidemic activity. To date, more than 150 triterpenes have been identified from the fruiting bodies, spores and mycelia of *G. lucidum* [38]. 

### 3.1. Cytotoxic and Antitumour Activity

The typical triterpenes isolated from *G. lucidum* together with their molecular structures, cytotoxic and anti-tumour action are presented in Table 2. 

It is more than obvious that the triterpene structure of ganoderic acids plays a vital part in their biological action. The activity of ganoderic acids could be mainly related to the hydroxylation of their lanostane triterpene structure. The active ganoderic acid A (GA-A) is hydroxylated at positions 7 and 15, while ganoderic acid H (GA-H) is hydroxylated at C-3, and the non-active ganoderic acid F (GA-F) is not hydroxylated (Table 2). Additional triterpenes with hydroxyl or acetoxy groups at locations 3, 7 and/or 15, such as ganoderic acid C1 (GA-C1), ganoderic acid C2 (GA-C2) [3], ganoderic acid D (GA-D) [39], ganoderic acid T (GA-T) [40], ganoderic acid X (GA-X) [41], ganoderic acid Y (GA-Y) [42], ganoderiol A [39], ganoderol B [42,43], lucidumol B and ganodermadiol [35,39], were also proven as inhibitors [44]. 

Ganoderic acid T caused a decrease in the proliferation of some cancer cells. It had higher cytotoxicity towards the 95-D lung cancer cell line than to normal cell lines. However, the effects of GA-T on human hepatoma SMMC-7721 cell lines and hepatic leukaemia factor (HLF) are similar. This indicates that GA-T has different cytotoxic potency against different tumour cells. The viability of 95-D cells was suppressed by 70% at 50 μg/mL at 24 h by GA-T. Ganoderic acid T at low concentrations could also strongly inhibit the formation of cell colonies of 95-D [40]. Results from Chen et al. [45] demonstrate that ganoderic acid T successfully inhibits cancer cell invasion in vitro and metastasis in vivo, and thus may act as a potential drug for cancer treatment.

Chen et al. studied the influence of ganoderic acid Me on tumour invasion. Results showed the anti-metastasis effects of ganoderic acid Me (GA-Me), which was proven by inhibition of cell adhesion and motility, as well as suppression of MMP2 and MMP9 gene expression. Thus, GA-Me could be a promising new anti-metastatic agent [46]. Ganoderic acid DM is a lanostane-type triterpene isolated from *G. lucidum* which shows cytotoxicity to cancer cell lines (PC-3 and LnCaP) [47,48].

Hsu et al. investigated the impact of lucidenic acids (A, B, C and N) on cell growth inhibition and apoptosis in human leukaemia cells HL-60. They discovered that lucidenic acid B lessened the cell capability of some tumour cell lines and encouraged apoptosis in HL-60 cells [49].

Gao et al. investigated the in vivo antitumour effects of the ganoderma alcohol, ganoderiol F, which exhibited the strongest activity in a cytotoxicity assay. It was administrated to Lewis lung carcinoma cell (LLC)-bearing mice at three doses of 5, 10, and 20 mg/kg/day. Ganoderiol F remarkably inhibited the tumour growth. Meanwhile, no obvious toxic or side effects were perceived [50].

Jiang et al. showed that ganodermanontriol, an alcohol present in *Ganoderma*, blocked proliferation and development of invasive, metastatic, and therapy-resistant human breast cancer cells. Ganodermanontriol blocked countenance of the cell cycle regulatory protein CDC20 [59]. 

### 3.2. Anti-Oxidative Effect

Free radicals and reactive oxygen species, which are produced as side-products of several metabolic processes, can seriously harm cells through oxidation processes. The long-term presence of free radicals and reactive oxygen species accelerates aging and numerous age-associated illnesses [60]. Some studies show that *G. lucidum* extracts increase the activity of super oxide dismutase and catalase, enzymes involved in eliminating damaging reactive oxygen species [61,62].

Zhu et al. studied the antioxidative activity of *G. lucidum* in combination with in vitro tests. The crude *Ganoderma* matter was exposed to boiling water media, afterwards the aqueous extract was separated. Terpene and polysaccharide rich fractions have been attained. Both of the fractions were analysed for their antioxidative effect. It has been demonstrated that the terpene fraction had the highest antioxidant activity. In that fraction, ganoderic acids A, B, C and D, lucidenic acid B and ganodermanontriol were present in highest proportion [63].

Heleno et al. concluded that extracts obtained from *G. lucidum* grown on germinated brown rice (GLBR) show important antioxidant activity against several antioxidant systems in vitro. Consumption of GLBR extract could pointedly increase the activity of some enzymes like superoxide dismutase, catalase, glutathione peroxidase in the sera, liver and brain of mice [64]. 

### 3.3. Anti-HIV Activity

HIV is a highly contagious virus affecting millions of people all over the world. HIV causes acquired immunodeficiency syndrome (AIDS). Present treatment approaches for HIV postpone the development of AIDS [65]. It has been demonstrated that various compounds from *G. lucidum* exhibit inhibitory effects on HIV progression. Isolated triterpenoids (ganoderic acid beta, lucidumol B, ganodermanondiol, ganodermanontriol and ganolucidic acid A) have been shown to have significant anti-human immunodeficiency virus (anti-HIV)-1 protease activity, with IC_50_ values of 20–90 microM [66]. In one of the earliest studies, El-Mekkawy et al. successfully isolated thirteen compounds from *G. lucidum* that had strong inhibitory activity against HIV-1 proteases [37]. Laccases, present in *G. lucidum* have been proven to inhibit HIV-1 reverse transcriptase [67]. A lot of study still needs to be done to establish a basis for *G. lucidum* isolates as anti-HIV agents, but nevertheless, triterpenoids seem to be the main class of compounds with anti-HIV effects.

### 3.4. Neuro-Protective Effects

Research on the harmful effects of oxidation in the human body has recently become a topic of great attention. Oxidative stress is one reason for the progression of many neurodegenerative illnesses, like Parkinson’s disease, Huntington’s disease, amyotrophic lateral sclerosis and Alzheimer’s disease (AD). One of the approaches for treating AD is to control the function of the neurotransmitter acetylcholine in the brain through the inhibition of acetylcholinesterase (AChE) [5]. Zhang et al. reported that the mixture of triterpenoid compounds in *G. lucidum* promoted neuronal survival and reduced fatigue [67]. The potential use of *G. lucidum* for neurological disease treatment has been studied, and it has been proven that continuing intake of *G. lucidum* can cut the progression of Alzheimer’s disease [67].

Inhibition of acetylcholinesterase (AChE) has been studied for *G. lucidum* water extracts and extracts obtained with supercritical fluid extraction. The AChE inhibition due to the addition of supercritical CO_2_ (SC-CO_2_) *G. lucidum* extracts and polysaccharidic water extracts was up to 22.54% [5]. Hasnat et al. studied the AChE inhibitory activity in water extracts. The authors reported 50% inhibition of AChE using crude hot water extracts [64]. The inhibition is quite high, probably due to the presence of polysaccharides, phenolic compounds and flavonoids, which are all known to inhibit AChE in high manner [68]. However, the SC-CO_2_ extracts were not further fractionated to determine the inhibitory effect of specific fractions, so the inhibitory activity of terpenoids cannot be confirmed.

## 4. Bioactivity of *G. lucidum* Polysaccharides

Polysaccharides are long-chain sugar molecules linked together by glycosidic bonds. Various types of them have been recognized in the *G. lucidum* tissue. Nevertheless, of the molecular weight of the polysaccharides, most have a positive influence on decreasing cancer development [6]. Structurally, the polysaccharides of *G. lucidum* mostly comprise high molecular weight heteropolymers, where the major component is glucose, but also including xylose, mannose, galactose, and fructose [69]. 

### 4.1. Cytotoxic and Antitumour Activity

To date, more than 200 different polysaccharides have been isolated from *G. lucidum* fruit bodies, spores, and mycelia or from liquid cultures. Even though selection of a particular extraction method depends on the structure of the cell wall, the most recently utilized extraction solvent has been hot water whereby water-soluble polysaccharides in particular have been isolated. The water-insoluble ones have been isolated by altering the pH of the solution. Those isolated compounds include β-d-glucans, α-d-glucans, α-d-mannans and polysaccharide-protein complexes [2]. The major bioactive polysaccharides classes are β-1-3 and β-1-6-d-glucans [70]. Polysaccharides named GTM1 to GTM6 were isolated successively from the mycelium of *Ganoderma* matter by Peng et al. The results demonstrated that GTM1 and GTM2 had a protein content of 13.5% and 20.1%. 

Isolated polysaccharides increase anti-tumour immune responses by motivating the activity of natural killer cells and cytotoxic T-lymphocytes [71]. In addition, the polysaccharides are also recognized to improve expression of the major histocompatibility complex in a melanoma cell line, which improves antigen exhibition and consequently stimulates viral and cancer resistance [70].

Stepwise precipitation or preparative gel permeation chromatography have been employed to yield polysaccharides with different molecular weights consisting of various monosaccharides. 

The biological activity of glucans is determined by their solubility in water, molecular weight and size, conformation and shape. *G. lucidum* polysaccharides (GLPS) exhibit the ability to improve the immune system and act as anticarcinogens. GLPS have been recognized as bioactive components, indicating several pharmacological properties [71].

The quality of immune system is crucial in cancer treatment. Glycoproteins, heteropolysaccharides and ganoderans A, B and C have high molecular weights, a hydrophilic character and high anti-tumour activity. Hydrophobic polysaccharides also possess anti-tumour activity [72]. Differences in molecular weights do not show the direct connection to the anti tumour activity. It has been assumed that large molecular weight polysaccharides have better anti-cancer mechanism since they can form several bindings to receptors or proteins due to the larger chains. The efficiency of β-glucans also depends on the number of lateral branches in the main chain, the length of the lateral chain and the ratio of the number of bonds [72]. Nonetheless, numerous low molecular weight polysaccharides possess considerable anti-cancer activities too. Beside molecular weight, the degree of branching, conformation, like triple helix, single helix, and random coil structures, are the factors that induce the highest antitumour activities of polysaccharides.

Different roles of polyscachharides in exerting antitumour effect include cancer-preventing activity, enhancement of immunity and direct antitumour activity to induce the apoptosis of tumour cells [73]. Polysaccharides usually do not act directly on cytotoxicity in the tumour cells, but exert anti-tumour activities through an enhancement of host-mediated immunity. It was reported that the amount of bioactive water-insoluble polysaccharides was greater than that of water-soluble polysaccharides [72,74]. 

Some researchers have demonstrated that water soluble GLPS with similar structures significantly inhibited plaque formation in the herpes simplex virus HSV-1 and HSV-2 [75,76,77].

Cao et al. described a GLPS consisting of d-rhamnose, d-xylose, d-fructose, d-galactose, d-mannose, and d-glucose in different molar ratios, which are linked together by β-glycosidic linkages. They found that this kind of polysaccharide can promote not only the maturation of cultured murine bone marrow-derived dendritic cells in vitro, but also the immune response initiation induced by dendritic cells [78].

In vivo and in vitro studies have proven that B lymphocytes, T lymphocytes, dendritic cells (DCs), natural killer cells (NKs) and mononuclear phagocyte cells are responsible for generating anti-tumour immune responses [71]. The polysaccharides GTM1, GTM2 and GTM3 showed significantly higher antitumour activity against the solid tumour sarcoma 180, with an inhibition ratio above 50% [79].

According to the latest studies by Wiater et al., α-d-glucans found in the cell walls of *G. lucidum* exhibit cytotoxic action in relation to human epithelial HeLa cancer cells [80]. 

Several in vivo studies have demonstrated that polysaccharides (β-d-glucans, heteropolysaccharides and glycoproteins) isolated from *G. lucidum* demonstrate antitumour activity against sarcoma 180 in mice. The stimulation of the immune system, which is mediated by polysaccharides, is thought to be the major mechanism of the antitumour action of *G. lucidum*. Among numerous polysaccharides, the β-d-glucans are mainly responsible for the antitumour effects [81].

A proteoglycan with a carbohydrate:protein ratio of 11.5:1 has been isolated by Zhao and co-workers. GLIS polysaccharide, as it is commonly termed, stimulates the proliferation of mouse spleen lymphocytes. This results in an increase of the amount of B cells and stimulated mouse spleen lymphocytes [82].

Another in vivo study has been carried out by Zhu et al. The fefficacy of GLPS on immunological effector cells, which play a key role against tumour progress under immunosuppression, was studied. The aim was to determine the in vivo efficacy of GLPS in improving the action of immunological effector cells in immunosuppressed mice. Mice were treated with a single dose of cyclophosphamide (Cy) (300 mg/kg) on the first day. 24 h later they were separated into groups. It was demonstrated that receiving Cy injection with 2.5 mg/kg, 25 mg/kg, and 250 mg/kg of Cy once per day for 7 days resulted in phagocytosis and cytotoxicity of macrophages [83]. No side effects were observed.

Li et al. have shown that GLPS can inhibit tumour growth in S180 ascitic tumour-bearing mice [84]. In a study by Gao et al., thirty-four advanced-stage cancer patients were included. Patients were treated with 1800 mg of Ganopoly^®^, three times daily, orally before meals for 12 weeks. Treatment with Ganopoly^®^ gave beneficial results. Afterwards a significant increase of interleukin (IL-2), IL-6, and interferon (IFN)-γ in plasma has been noted. The levels of IL-1 and tumour necrosis factor (TNF-α) were significantly decreased. The amount of clusters of differentiated protein cells was considerably increased after 12 weeks of treatment with Ganopoly^®^. Amongst them, the quantity of CD3^+^, CD4^+^, and CD8^+^ were just slightly enlarged. CD4:CD8 T cell proportions remained similar. This study suggests that Ganopoly^®^ can improve the immune responses in patients with progressive-phase cancer [85].

In vivo tests on mice proved antitumour activities and an acceptable tolerability after oral ingestion of GLPS in many tumour strain lines (Ca755, s/c P388, s-180) [86]. GLPS showed positive effects in combination with chemotherapeutic drugs. Theraphy of S180 ascitic tumour-bearing mice with β-(1→6)-branched β-(1→3) glucohexaose, obtained from GLPS, not only greatly increased the inhibition of S180 by the chemotherapeutic agent cyclophosphamide (CPA), but also lessened the injuries caused by CPA [87]. 

### 4.2. Anti-Oxidative Effect

Determination of in vitro antioxidant activity has been carried out by several different methods such as 2,2-diphenyl-1-picrylhydrazyl (DPPH) scavenging activity, reducing power, chelating ability, hydroxyl radical scavenging activity, 2,2′-azino-bis(3-ethylbenzothiazoline-6-sulphonic acid (ABTS) scavenging activity, superoxide radical scavenging activity and hydrogen peroxide scavenging activity. All of them have confirmed the capable radical scavenging abilities of polysaccharides and polysaccharide-complex isolated from different parts of the crude *G. lucidum*. Despite the great antioxidant potential of homo-glucans and hetero-glucans from this species, their underlying mechanism of action has not yet been systematically elucidated [88]. 

Chemical composition, molecular mass, type of glycosidic linkage and conformation are the main factors affecting the bioactivity of polysaccharides. Among them, molecular weight was one of the most important structural features of polysaccharides, as it is related to the number of reductive hydroxyl group terminals (on a per unit mass basis) responsible for accepting and eliminating free radicals. Low molecular weight polysaccharides would therefore have proportionally higher antioxidant ability (GLP_L_1 and GLP_L_2) [89]. The scavenging effect against superoxide radicals of low molecular weight chitosan (9 kDa) was more potent than that of high molecular weight chitosan (760 kDa) [90]. Structural analyses of *G. lucidum* polysaccharides (GL-PSs) indicate that GL-PSs are heteropolymers, in which glucose occurs as the major sugar component, while xylose, mannose, galactose and fucose are present in lower amounts and in different conformations, including 1–3, 1–4, and 1–6-linked β and α-d (or l)-substitutions [20].

Zhu et al. showed the capacity to affect the immune system of low-molecular-weight polysaccharides contained in water extracted from the fruit bodies of *G. lucidum* [91]. Polysaccharides isolated from the fruit bodies of Reishi showed antioxidant activity [92]. Kao et al. 2011 reports that β-1,3-glucan (a low-molecular weight glucan) isolated from *G. lucidum* was able to significantly increase (from 40% to 80%) the viability of a mouse leukaemic monocyte macrophage cell line (RAW 264.7) with H_2_O_2_-induced oxidative stress, and reduced reactive oxygen species (ROS) formation. It also suppressed the activities of neutral and acidic sphingomyelinases (SMases) [93]. Mannose-based homo-polysaccharide was able to increase the activity of antioxidant enzymes. In addition to the antioxidant ability of high-purity polysaccharides, some studies highlight the high radical scavenging effect of polysaccharide conjugates such as polysaccharide-protein complexes and polyphenolic-associated polysaccharides, metal ion-enriched polysaccharides, polysaccharide chelating metal and polysaccharide mixtures [89]. Liu and co-workers investigated the relation between the protein or peptide moiety in polysaccharides and the scavenging effect on superoxide and hydroxyl radicals [94]. Polysaccharide-protein complexes extracted from *G. lucidum* with lower polysaccharide/protein ratios were more effective in the scavenging function. The antioxidant activity of polysaccharide-protein complexes attained by ultrasound-assisted extraction was generally higher than with the conventional hot-water method, which can probably be attributed to the fact that ultrasound treatment produced an increase in the protein content in polysaccharides. Further studies indicated that, besides the quantity of protein or peptide molecules, their composition has to be taken into consideration. Amino acids, such as tyrosine, methionine, histidine, lysine and tryptophan, are capable of donating protons to electron-deficient radicals. 

The most abundant polysaccharide isolated from *G. lucidum*, GLP, consists of 14 amino acids. Several poysaccharides (d-rhamnose, d-xylose, d-fructose, d-galactose, d-mannose and d-glucose) are present as sugars. The molecule has a strong ability to intensify antioxidants, serum insulin level and to decrease lipid peroxidation. The survival rate of macrophages, and protecting the mitochondria against injury by membrane-permeant oxidant (*t*-BOOH) [95] has also proved the high antioxidant activity.

In the presence of oxidation substrates such as plant oils, the antioxidant activity of GLP is comparable to that of synthetic antioxidant butylated hydroxytoluene (BHT) in soybean oil, which blocks soybean lipoxygenase activity [96].

Proteoglycan (GLPG), *G. lucidum* immunomodulation substance (GLIS), a water-soluble glycopeptide (PGY), polysaccharide peptide (GL-PP), and a fucose-containing glycoprotein fraction (F3) have also been isolated from different parts of *G. lucidum* [20].

The chelating ability of polysaccharides depends on the presence of uronic acid and sulphate groups. However, carboxymethylated polysaccharide (C-GLP) from *G. lucidum* showed only a weak antioxidant effect [97]. 

Another approach to influencing the antioxidant activity of polysaccharides to some extent is chemical modification by moderating the solubility of water-insoluble polysaccharide. Chen et al. reported that *G. lucidum* polysaccharides could significantly enhance antioxidant enzyme activities [92]. Furthermore, Liu et al. proved that sulfation effectively enhanced the water solubility and bile acid-binding capacities of a water-insoluble polysaccharide from *G. lucidum* (GLP) [98].

### 4.3. Neurological Effects

Polysaccharides isolated from *G. lucidum* are also believed to confer a neurological benefit. It has been known from ancient times that *G. lucidum* acts as an analgesic and has relaxing properties [67]. Matsuzaki et al. have reported that the water extract rich with polysaccharides exhibits an anti-depressant-like effect and decreases anxiety-type behaviour in rats [99].

### 4.4. Antimicrobial Effects

GLPS are mostly known as antitumour agents; therefore, only a few reports have been published on the antimicrobial activities of polysaccharides from *Ganoderma* species. Moreover, most studies have been carried out in vitro. The mechanisms of the antimicrobial and antiviral activities of *Ganoderma* remain largely undefined. Although the extracts contain a number of biologically active compounds (carbohydrates, glycosides, triterpenoids, phenolic compounds and tannins) that exert a certain degree of antimicrobial activity, principally in a mixture, antibacterial activity is partially derived from the inhibiting capability of some polysaccharides present in *G. lucidum*. Given the presence of a broad spectrum of antimicrobial agents, extracts could inhibit Gram-positive as well as Gram-negative bacteria. Therefore, it is not surprising that the majority of research has been performed on extracts from the fruiting body and mycelium and a few on the activity of isolated polysaccharides. Generally, *G. lucidum* aqueous and organic solvent (hexane, dichloromethane, ethyl acetate and methanol) extracts act against *Bacillus cereus, Enterobacter aerogenes, Staphylococcus aureus, Escherichia coli* and *Pseudomonas aeruginosa*. The extraction method is closely related to the inhibitory action of the extracts against certain microorganism strains. According to Kamra and Bhatt, a water extract of the *G. lucidum* fruit body was equally inhibitory against all the tested strains (*Pseudomonas aeruginosa, Proteus vulgaris* and *Enterococcus faecalis*) except for *Listeria* monocytogenes. A moderate inhibitory effect was noted against *Salmonella typhimurium, Klebsiella pneumoniae* and *Streptococcus mutans*, and the least effect against *Bacillus subtilis*. Hexane, dichloromethane and ethyl acetate show low solvatation power for the isolation of antimicrobial agents from the tissue [100]. Heleno et al. reported that methanolic extract showed higher activity against *S. aureus* and *B. cereus* than the antibiotics ampicillin and streptomycin, whereas *S. aureus* and *B. cereus* were the most susceptible bacteria. Minimal inhibitory concentrations were in the range of 0.0125–0.75 mg/mL and bactericidal concentrations of 0.035–1.5 mg/mL. However, the ability against *P. aeruginosa* was the weakest [101]. 

Polysaccharides obtained from *G. lucidum*, in which d-glucose is usually the major component, have a strong participation in inhibiting the growth of mainly pathogenic bacteria. Polysaccharide species isolated from the strains of the *G. lucidum* fruiting bodies and those obtained from various sawdust cultivation substrates showed the highest inhibitory ability towards *M. luteus* (MIC 0.62 or 1.25 mg/mL) [102]. Extraction with hot water from cultivated *G. lucidum* and further fractionation by ethanol precipitation/DEAE-cellulose column chromatography gave polysaccharides inhibiting the growth of plant pathogens (*Erwinia carotovora*, *Penicillium digitatum*, *Botrytis cinerea*) and five harmful food-microorganisms (*Bacillus cereus*, *Bacillus subtilis*, *Escherichia coli*, *Aspergillus niger* and *Rhizopus nigricans*) [103]. GLPS show only a weak capacity to inhibit the growth of two microorganisms commonly present in food: *E. coli* and *A. niger. G. lucidum*, even though water is the most common medium for extraction of polysaccharides. Polysaccharides obtained from *G. lucidum* fruiting bodies, containing either β-1,3-glucans or α-1,4-linked polymannose are generally responsible for their biological activity, and can act as in vivo agents. Exopolysaccharide (EPS) obtained from *G. lucidum* showed the highest potential against the growth of *B. cereus*, among other bacterial species (23 ± 0.61 mm and 18 ± 0.38 mm, respectively) [88]. Exopolysaccharide contains several high-molecular weight compounds and possesses both adsorptive and adhesive properties due to the presence of various charged groups. Antibacterial activity of EPS from a basal medium and a malt medium against some bacterial species has been evaluated. EPS from both media showed the highest activity against the growth of *Bacillus cereus* (23 ± 0.61 mm and 18 ± 0.38 mm), respectively [104]. 

## 5. Conclusions

It has been proven that *G. lucidum* contains a wide variety of bioactive components that promote several beneficial effects on health. Consequently, most studies to date have focused on this class of compounds. The structural variability of these isolated compounds shows varying capacity for carrying biological information. In the present review, the antitumour, antimicrobial, antioxidant and antiacetylcholinesterase effects of isolated compounds from *G. lucidum* have been taken into consideration. There are two main groups, triterpenes and polysaccharides, that have been researched in detail. Triterpenoids have been reported as having anti-hypertensive, hypocholesterolemic, hepatoprotective and anti-histaminic effects, along with antitumour and anti-angiogenic activity. 

Antioxidant, antitumour and antibacterial potential of polysaccharides has been demonstrated by both in vitro and in vivo studies. All of the mentioned activities are related to polysaccharide molecular weight, level of branching and water solubility. Over the last three decades, many polysaccharides from *Ganoderma* species have been extracted utilizing various methods using different solvents. The most common polysaccharides isolated from *G. lucidum* are α- or β-(1→3)-, (1→6)-glucans and hetero-polysaccharides, with different mixtures of sugars with different molecular weights. There is a general lack of data regarding the antimicrobial activity of *G. lucidum* isolated compounds. Some polysaccharides show antibacterial activity, inhibiting bacterial growth or inducing the death of pathogenic bacteria.

Due to the lack of results, intense investigation should still be performed in the field (e.g., human clinical trials). Up till now, the available data suggests that *G. lucidum* has a high potential to be accepted as a good health food supplement for patients experiencing cancer therapy. The total chemical evaluation of polysaccharides is particularly significant for development of an appreciative knowledge of the main features responsible for their powerful action. That knowledge and further investigation could facilitate the development of new nutraceuticals and pharmacological formulations.

## Figures and Tables

**Table 1:** Other substances of *G. lucidum* [22].

Compound	Content [mg/100 g]
Calcium	832
Phosphorus	4.150
Iron	82.6
Magnesium	1.030
Sodium	375
Potassium	3.590
Vitamin B1	3.49
Vitamin B2	17.10
Vitamin B6	0.71
Choline	1.150
Niacin	61.9
Inositol	307

**Table 2:** Triterpenes isolated from *G. lucidum*.

Triterpene		Tumour cells	Action	Molecular Structure	Target	Ref.
Ganoderic acid (3α,22β-diacetoxy-7α-hydroxyl-5α-lanost-8,24-*E*-dien-26-oic acid)	in vitro	Lung:95D Cervical: HeLa	cytotoxic	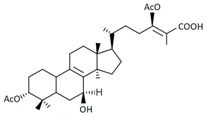		[36]
Ganoderic acid Mk	in vitro	Lung:95D Cervical: HeLa	cytotoxic	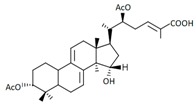		[36]
Ganoderic acid S		Lung:95D Cervical: HeLa	cytotoxic	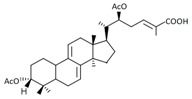		[36]
Ganoderic acid Mf		Lung:95D Cervical: HeLa	cytotoxic	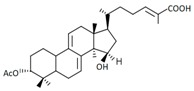		[36]
Ganoderic acid R		Lung:95D Cervical: HeLa	Cytotoxic	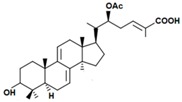		[36]
Ganoderic acid Mc		Lung:95D Cervical: HeLa	Cytotoxic	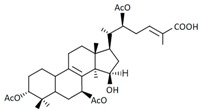		[36]
Ganoderic acid A		Breast: MDA-MB-231	Inhibited growth and invasive behaviour of breast cancer cells	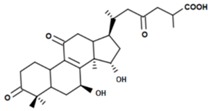	AP-1 or NF-κB	[44]
Ganoderic acid F		Breast: MDA-MB-231	Ineffective	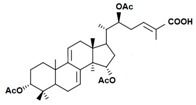	AP-1 NF-κB uPA Cdk4	[44]
Ganoderic acid H		Breast: MDA-MB-231	Inhibited growth and invasive behaviour of breast cancer cells	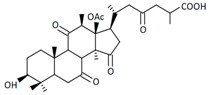	AP-1 NF-κB uPA Cdk4	[44]
ganoderic acid X	in vitro	Liver: HuH-7 Colon: HCT-116	Inhibits topoisomerases and induces apoptosis of cancer cells	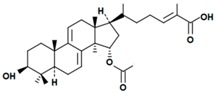	ERK, JNK	[41]
ganoderic acid T	in vitro	lung: 95D liver: SMMC7721 epidermis: KB-A-1 and KB-3-1 cervix: HeLa	Decrease in proliferation of some cancer cells. Strongly inhibits the formation of cell colony of 95-D.	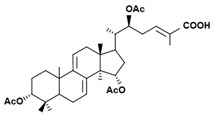	p53 Bax caspase-8	[40]
	in vitro	colon: HCT-116	Inhibits proliferation		NFκB-α, MMP-9, uPA, iNOS	[45]
	in vitro	melanoma: A375 colon: Ls174t	Inhibits growth		MMP2/9 NF-κB	[51]
	in vivo	Lung: LLC	Suppresses tumour growth and LLC metastasis		MMP 2/9	[45]
ganoderic acid Me	in vitro	Colon: HCT-116 HCT-8	Possesses cytotoxicity	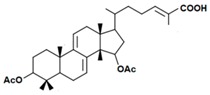	p53 Bax	[52,53]
	in vitro	Lung: 95-D	inhibits cancer cell invasion		MMP 2/9	[46]
	in vitro in vivo	Breast: MDA-MB-231	inhibits proliferation and invasion and induces apoptosis		NF-κB, TNF-α, VEGF, IL-6/8, MMP-9, Bcl-2, c-Myc and CCND1	[54]
ganoderic acid D	in vitro	Cervical: HeLa	inhibits proliferation	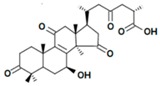	AHA1 Cytokeratin 19 Cytokeratin 1 PRDX3	[55]
ganoderic acid E	in vitro	Liver: Hep G2 Hep G2,2,15	cytotoxic	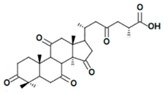		[56]
ganoderic acid DM	in vitro	Prostate: PC-3, LnCaP	inhibits prostate cancer cell proliferation and metastasis	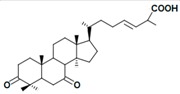	MMP-2 MMP-9 IL-1, IL-6, TNF-α, and CCL-2/MCP-1	[48]
lucidenic acid A		leukaemia: HL 60	decreases cell population growth, cell cycle arrest	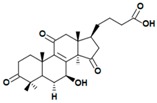	Bcl-2 caspase-9 caspase-3	[49]
lucidenic acid B		leukaemia: HL 60	Induces apoptosis	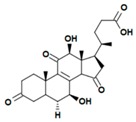	Bcl-2 caspase-9 caspase-3	[49,57]
		liver: HepG2 Lymphoma: CA46			MMP-9, NF-κB, ERK1/2, AP-1, c-Jun, c-Fos	
lucidenic acid C		leukaemia: HL 60	decreases cell population growth, cell cycle arrest	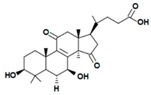	Bcl-2 caspase-9 caspase-3	[49]
lucidenic acid N		leukaemia: HL 60	decreases cell population growth, cell cycle arrest	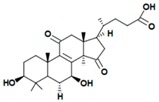	Bcl-2 caspase-9 caspase-3	[49]
Ganoderiol F	in vitro	Lung: LLC Meth A, sarcoma: Sarcoma-180 Carcinoma: T-47D	Cytotoxicity	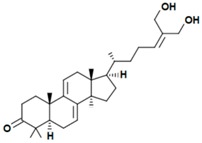		[58]
	in vivo	Lung: LLC	inhibitory effect on tumour growth			[50]
Ganodermanontriol		Colon: HCT-116, HT-29	Inhibition of cell proliferation	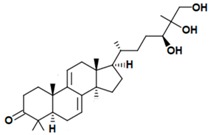	β-catenin, cyclin-D1, Cdk4, PCNA, E-cadherin	[59]
		Breast: MDA-MB-231			uPA, uPAR	

## Abbreviations

95D	Human lung cancer cell line
ABTS	2,2′-azino-bis(3-ethylbenzothiazoline-6-sulphonic acid
AC	Acetylcholine
AChE	Acetylcholinesterase
AD	Alzheimer’s disease
Aha1	Activator of HsP90 ATPase
AIDS	Acquired immunodeficiency syndrome
AP	Activator protein
Bax	Bcl-2-associated X protein
Bcl-2	B-cell lymphoma
BHT	Butylated hydroxytoluene
Capase-8	Capase protein
CCL-2	Carnival cruise lines
CD	Cluster of differentiation protein
CDC20	Cell cycle regulatory protein
CDK	Cyclin-dependent kinase
c-Fos	Protein code by Fos gene
C-GLP	Carboxymethylated polysaccharide
c-Jun	Protein code by Jun gene
c-MMC	Myelocytomatosis viral oncogene homolog
CPA	Cyclophosphamide
Cy	Cyclosporine
Cy775	Adenocarcinoma
CyclinD1	Protein by the CCND1 gene
DCs	Dendric cells
DPPH	2,2-diphenyl-1-picrylhydrazyl
EPS	Exopolysaccharide
ERK	Extracellular regulated kinase
GA	Ganoderic acid
GLBR	Germinated brown rice
GLIS	Ganoderma lucidum immunomodulation substance
GLPG	Ganoderma lucidum proteoglycan
GL-PP	Ganoderma lucidum polysaccharide peptide
GLPS	Ganoderma lucidum polysaccharides
GTM	Ganoderma water soluble polysaccharides
HCT-116	Human colon cancer cell line
HCT-26	Human colon cancer cell line
HeLa	Human cervical tumour cell line
Hep-G2	Human liver tumour cell line
HL 60	Human promyelocytic leukaemia cell line
HLF	Hepatic leukaemia factor
HSV	Herpes simplex virus
HTC-116/8	Human colon tumour cell lime
HuH7	Human hepatocarcinoma cell line
IFN	Interferon
IL	Interleukin
iNOS	Inducible nitrine oxide synthase
JNK	Jun nuclear kinase
LLC	Human lung cancer cell line
LnCaP	Human prostate cancer cell lines
MCP-1	Monocyte chemoattractant protein 1
MDA-MB-231	Human breast cancer cell line
MMP	Matrix metalloproteinase
NF-κb	Nuclear factor kappa beta
NFκB-α	Nuclear factor kappa beta – alpha
P388	Human leukaemia cell line
P53	Protein 53
PC-3	Human prostate cancer cell lines
PCNA	Proliferating cell nuclear agent
PGY	Water-soluble glycopeptide
PRDX3	Peroxiredoxin 3
RAW 264.7	Leukaemic monocyte macrophage cell line
S-180	Human sarcoma 180
SMMC-7721	Human hepatoma cell lines
T-47D	Human carcinoma cell line
TNF	Tumour necrosis factor
uPa	Urokinase-type plasminogen activator
uPAR	Urokinase-type plasminogen activator receptor
VEGF	Vascular endothelial growth factor
